# Clinical relevance of partial HPV genotyping in cervical cancer screening

**DOI:** 10.1038/s41598-026-36594-z

**Published:** 2026-02-04

**Authors:** Aarno Leino, Eero Numminen, Saara Kares, Markus Meriö, Laura Kotaniemi-Talonen, Ivana Kholová, Karolina Louvanto

**Affiliations:** 1 Department of Obstetrics and Gynecology, Faculty of Medicine and Health Technology, Tampere University, Tampere, Finland; 2 Department of Pathology, Fimlab Laboratories, Tampere, Finland; 3 Department of Obstetrics and Gynecology, Tampere University Hospital, Tampere, Finland; 4 Faculty of Medicine and Health Technology, Tampere University, Tampere, Finland; 5 Institute of Clinical Medicine, Pathology, and Forensic Medicine, University of Eastern Finland, Kuopio, Finland; 6 Department of Clinical Pathology, Diagnostic Imaging Center, Kuopio University Hospital, Kuopio, Finland

**Keywords:** HPV, Screening, Cervical cancer, HPV genotyping, Partial genotyping, Cancer, Diseases, Health care, Medical research, Microbiology, Oncology

## Abstract

Partial human papillomavirus (HPV) genotyping is increasingly used to triage high-risk (HR)-HPV-positive women in national cervical cancer screening. This study evaluated whether separate triage for HPV genotypes 16 and 18 could enhance the effectiveness of Finland’s program. Data from 76,482 women participating in primary HPV screening in Tampere and surrounding municipalities (2012–2023) were analyzed. Partial genotyping identified HPV16, HPV18, and 12 other HR-HPV-types, and the association between genotype and high-grade squamous intraepithelial lesions or worse (HSIL +) detection was assessed. Among 6031 HR-HPV-positive women, HSIL + prevalence was highest in HPV16-positive women (37.3%) followed by HPV18 (26.0%) and other HR-HPV types (20.3%). HSIL + detection declined with age: 26.2%(age < 45), 15.9%(age 45–50), and 11%(age > 50)(*p* < 0.001). HPV16 showed the highest persistence upon re-testing (69.6% single; 84.6% co-infections), compared to 53.8% for other HR-HPV types. Among women with NILM cytology, HPV16 infections conferred a significantly higher risk of HSIL + compared to infections with other HR-HPV types. This risk was evident for both single HPV16 infection (OR 2.41;95%CI:1.55–3.75) and HPV16 coinfections (OR 3.86;95%CI:2.14–6.96). These findings support the integration of age-specific strategies and partial HPV16/18 genotyping into Finland’s screening program. A refined triage model, including immediate colposcopy referral for HPV16/18-positive women, could improve patient management and screening efficiency.

## Introduction

The discovery of human papillomavirus (HPV) as the etiological agent of cervical cancer has revolutionized screening approaches, leading to the development of HPV testing^1–3^. Since 2010, countries worldwide have transitioned from cytology based screening to HPV primary screening^4,5^, which provides greater sensitivity for the detection of high-grade cervical disease^6–11^. Yet, this shift has introduced challenges related to decreased screening specificity and increased referral rates leading to excess follow-ups that bottleneck the healthcare system^12^. Furthermore, the high sensitivity of HPV testing raises concerns regarding the potential overdiagnosis of mild or regressive cervical lesions. To navigate these challenges and increase screening specificity, reflex cytology is commonly used to triage HR-HPV positive women^13^.

To address these limitations, the implementation of type-specific HPV genotyping in screening algorithms has been widely researched^14–18^. The strategy is based on varying oncogenic properties of different HPV genotypes^19–23^. The current screening assays offer either no genotyping, partial HPV genotyping distinguishing HPV16, 18 and the 12 other HR-HPV genotypes combined (HPV31, 33, 35, 39, 45, 51, 52, 56, 58, 59, 68 and 66), or extended genotyping with HPV16 and 18 and the remaining HR-HPV genotypes in groups or individually detected^24–26^. Consequently, several countries, including the Netherlands, Sweden, Norway, Denmark, and Turkey, have already shifted towards genotype-specific risk stratification in their primary screening programs^18,27–30^. Furthermore, international guidelines increasingly recommend leveraging genotyping information for management decisions^17,31^. For example HPV16/18-positive women are referred directly to colposcopy, while women with other HR-HPV types undergo additional triage or repeat testing^15,17,27,32^. Separate triage for different HPV genotypes further improves risk stratification. This approach is suggested to reduce unnecessary procedures while improving detection of high-grade cervical lesions^15,33^.

In Finland, HR-HPV positive women are currently triaged with reflex cytology to guide clinical management, resulting in either repeat testing in 12–24 months or immediate referral to colposcopy^5^. While HR-HPV-testing has been part of Finnish cervical cancer screening since 2012, partial genotyping information has not been incorporated into the risk stratification^34^. Given the heterogeneity in HPV prevalence and test performance across populations, population level research is essential before adopting new strategies^35^.

The aim of this study was to evaluate whether incorporating partial HPV genotyping into the triage of HR-HPV positive women could improve the effectiveness of Finland’s cervical cancer screening program.

## Material and methods

### Study cohort

This study evaluates 83,000 women that took part to the Finnish national cervical cancer screening in the city of Tampere and its’ surrounding municipalities (Kangasala, Lempäälä, Nokia, Pirkkala and Ylöjärvi) between the years 2012 to 2023. In Finland, organized screening is conducted every five years. Specific target age cohorts (women turning 30, 35, 40, 45, 50, 55, and 60 years old in the screening year) have traditionally been invited; with the 65-year-old cohort included since 2022.

In Tampere region, cervical cancer screening is organized by Fimlab Laboratories Ltd. (a publicly owned private service provider). Samples for HR-HPV testing and cervical cytology are collected simultaneously during the screening visit. Participants with negative HR-HPV tests were considered screening negative and returned to the routine 5-year screening interval. For HR-HPV positive women, reflex cytology triage was performed. Immediate colposcopy referral was triggered by cytology results showing a low-grade squamous intraepithelial lesion or worse (LSIL +). Conversely, women with cytology showing atypical squamous cells of undetermined significance (ASC-US) or negative for intraepithelial lesion or malignancy (NILM) were directed to repeat testing in 12–24 months^36^. Upon retesting, any persistent HR-HPV positivity warranted colposcopy referral, regardless of the new cytology result. Given the real-world nature of our screening data, clinicians occasionally referred women for colposcopy without meeting the national referral criteria. These cases were included for the analysis if the necessary information for the analysis was available. Screening test results and the clinical follow-up data of the HR-HPV positive women referred for colposcopy were retrieved from the Tampere university hospital patients’ charts. All women were followed up until the end of 2023.

### Ethics declarations

The study protocol has been approved by the ethical board of the Pirkanmaa wellbeing services county (R13094/25.03.2013 and R21034/31.03.2021). No individual informed consent was required, as the study was conducted within routine screening practice, and individual participant data were not recognizable. All methods were performed in accordance with the Finnish Medical Research Act, the Finnish Biobank Act and the Finnish Act on the Medical Use of Human Organs, Tissues and Cells.

### HPV genotyping and cervical cytology

During regular screening practice, HPV genotyping was performed at the central screening laboratory (Fimlab Laboratories Ltd) using the Abbot RealTime High Risk (HR) HPV array (2012–2020) and Roche Cobas 4800 HPV assay (2021–2023). Both arrays utilize polymerase chain reaction (PCR) to detect high risk HPV genotypes 16, 18 and other 12 high risk genotypes together (31, 33, 35, 39, 45, 51, 52, 56, 58, 59, 66 and 68). As per the screening protocol, cytological samples were examined only from the HR-HPV positive women and the pap smear cytology was graded according to the Bethesda System (TBS)^37^.

### Colposcopy referrals and punch biopsies

All colposcopies were performed at the gynecology outpatient clinic at Tampere university hospital, Finland. Punch biopsies were performed during the colposcopy and histological results were obtained. If high-grade squamous intraepithelial lesions (HSIL) were detected, a loop electrosurgical excision procedure (LEEP) was performed. All women were followed according to the Finnish current care guidelines of cervical precursors and cancer^38^.

### Statistical analysis

The study included all screened women with at least one negative or positive HR-HPV result. Women with only cytological or histological result without HPV-result were excluded. Only one screening round was included for each woman. For women screened multiple times between 2012 and 2023, the first screening round with positive screening result was included. Cytology results were included in the analysis if they corresponded to a positive HR-HPV test and only the most severe cytology result was included, in case there were multiple samples taken at the same time. For the analysis, atypical squamous cells cannot exclude HSIL (ASC-H) cytology results were merged with HSIL cytology, and atypical glandular cells not otherwise specified (AGC-NOS) and atypical glandular cells, favor neoplasia (AGC-FN) were merged. Histological findings HSIL, adenocarcinoma in situ (AIS) and carcinomas were merged as HSIL + for the analysis.

To allow comparisons by age, women were divided into three different invitation age groups: (1) youngest age group consisting of women invited to screening at the age of 30, 35 and 40; (2) women invited at age 45 and 50; and (3) the oldest age group of women including those invited at age 55 and 60 and 65. Cytology results and histological findings among women positive for HPV16, 18 and/or the 12 other HR-HPVs combined was assessed. Logistic regression was used to calculate the odds ratios (OR) with 95% confident intervals (CIs) for the associations of different HR-HPV genotype groups with HSIL or worse histology. The Chi squared test was used to test the difference between different HR-HPV genotype groups and age groups. All statistical analyses were performed using STATA17.0 (STATA Corp., TX, USA) and two-sided *P*-values < 0.05 were considered statistically significant.

## Results

### Screening outcomes and baseline characteristics

**Fig. 1:**
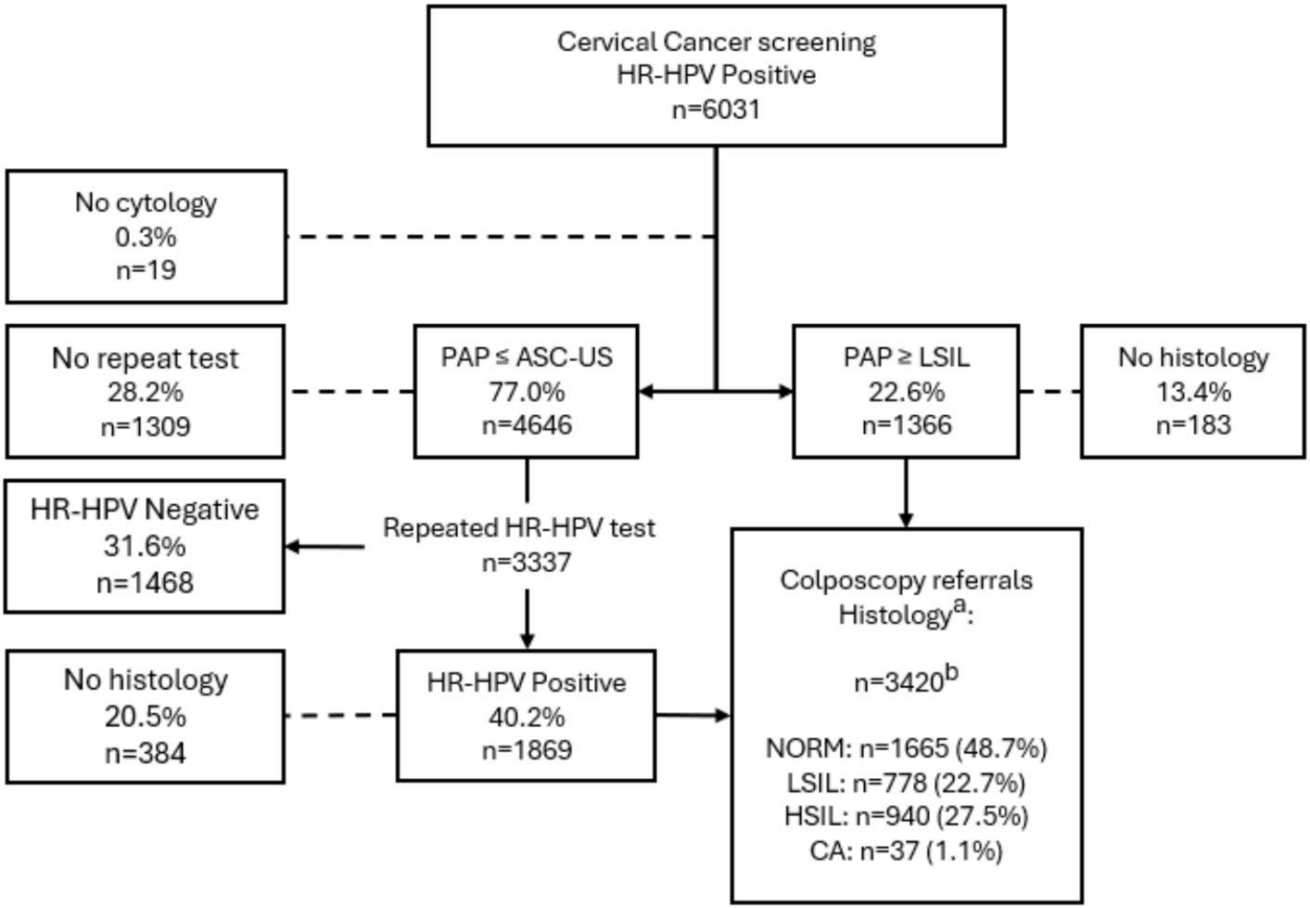
Flow chart of the 6031 HR-HPV positive from the initial screening round from the Finnish national cervical cancer screening between the year of 2012 to 2023 in the Tampere region and surrounding municipalities. Screening is offered in Finland to all women between the ages of 30 to 65 years old with a five-year screening interval. Footnote: ^a^Punch biopsy or LEEP confirmed histology. ^b^Number includes 1485 cases from the HR-HPV Positive group, 1183 cases from the PAP ≥ LSIL group and 752 endpoint histology cases from colposcopy referrals not meeting the national referral criteria from “No cytology” (n = 5), “No repeat test” (n = 625) and “HR-HPV Negative” (n = 122) -groups that were used in the analyses. Abbreviations: HR-HPV = high-risk human papilloma virus, PAP = Pap cytology, ASC-US = atypical squamous cells of undetermined significance, LSIL = low-grade squamous intraepithelial lesion, NORM = normal tissue, HSIL = high-grade squamous intraepithelial lesion, CA = carcinoma, LEEP = loop electrosurgical excision procedure.

Between 2012 and 2023, 76 482 of the 83 000 women were screened with primary HR-HPV-test, with 6031 (7.9%) resulting HR-HPV positive at baseline (Fig. 1). Of these, 1366 (22.6%) were referred directly for colposcopy due to LSIL + cytology, while 4646 (77.0%) with NILM or ASC-US cytology were scheduled for retesting at 12 to 24 months. Among those with NILM or ASC-US cytology, 1309 (28.2%) were either lost to follow-up, protocol violators or referred directly to colposcopy. Of the 3337 women who participated in HPV retesting, 1869 (56.0%) remained HR-HPV positive and were subsequently referred for colposcopy. After adding the 752 women referred to colposcopy without meeting the national referral criteria, altogether 3420 (56.7%) of the 6031 women attended a colposcopy appointment and a histological sample was taken during the one screening round. The mean follow-up time of the women referred to colposcopy was 3.4 years (± SD 2.3) ranging from six months to 10 years and 11 months. For the 6031 initially HR-HPV positive women, including the women not referred to colposcopy, the mean follow-up time was 3.0 years (± SD 2.4).

The mean age of HR-HPV positive women was 42.9 (± SD 10.0) years, and by HR-HPV genotypes the mean ages among the HPV16, 18 and other HR-HPV positive women were 40.8 (± SD 9.7), 41.8 (± SD 9.7) and 43.5 (± SD 10.0) years, respectively.

### Genotype and cytology association with HSIL+ 

Cytological results and histological outcomes differed across the HR-HPV groups (Table 1). Notably, HPV16 and 18 were associated with a higher proportion of histological HSIL + compared to the other HR-HPV types. With HPV16 single or co-infection with other HR-HPV the HSIL detection ranged from 43.4% (n = 227/523) to 53.3% (n = 112/210) compared to the group of other HR-HPV types with HSIL detected in 21.6% (n = 521/2412). The same trend was seen also with cervical cytology, where 17.5% (n = 139/794) to 22.5% (n = 65/289) of women with HPV16 alone or combined with other HR-HPV had HSIL/ASC-H cytology in comparison to 6.2% (n = 278/4486) with those with other HR-HPV only.

**Table 1:** Characteristics of the 6031 HR-HPV-positive women from the initial Finnish national cervical cancer screening round between the year of 2012 to 2023 in Tampere and surrounding municipalities^a^. Partial HPV genotyping for 16, 18 and other high-risk (othr) HPVs (including 12 HR-HPVs of 31, 33, 35, 39, 45, 51, 52, 56, 58, 59, 66, 68) was done.

HPV genotype n (%)								
	Single 16	Single 18	16/18	16 + othr	18 + othr	16/18 + othr	othr	Any HPV
*Invitation age group (years)*^b,c^								
30–40	496 (62.3)	179 (59.7)	11 (64.7)	186 (64.4)	65 (59.6)	14 (70.0)	2294 (51.0)	3245 (53.8)
45–50	162 (20.4)	66 (22.0)	3 (17.6)	60 (20.8)	26 (23.9)	4 (20.0)	1145 (25.4)	1466 (24.3)
55–60	138 (17.3)	55 (18.3)	3 (17.6)	43 (14.9)	18 (16.5)	2 (10.0)	1061 (23.6)	1320 (21.9)
*Pap cytology*^b^* (n = 6012)*								
NILM	383 (48.2)	151 (50.7)	4 (23.5)	103 (35.6)	48 (44.4)	4 (20.0)	2702 (60.2)	3395 (56.5)
ASC-US	162 (20.4)	50 (16.8)	5 (29.4)	57 (19.7)	23 (21.3)	5 (25.0)	949 (21.2)	1251 (20.8)
LSIL	67 (8.4)	23 (7.7)	2 (11.8)	50 (17.3)	15 (13.9)	3 (15.0)	427 (9.5)	587 (9.8)
HSIL/ASC-H	139 (17.5)	26 (8.7)	3 (17.6)	65 (22.5)	12 (11.1)	4 (20.0)	278 (6.2)	527 (8.8)
AGC-NOS/AGC-FN	43 (5.4)	48 (16.1)	3 (17.6)	14 (4.8)	10 (9.3)	4 (20.0)	130 (2.9)	252 (4.2)
Carcinoma	0 (0.0)	0 (0.0)	0 (0.0)	0 (0.0)	0 (0.0)	0 (0.0)	0 (0.0)	0 (0.0)
*Histology*^b^* (n = 3420)*								
norm	204 (39.0)	80 (46.2)	5 (45.5)	55 (26.2)	35 (47.3)	3 (17.6)	1283 (53.2)	1665 (48.7)
LSIL	77 (14.7)	33 (19.1)	2 (18.2)	39 (18.6)	21 (28.4)	3 (17.6)	603 (25.0)	778 (22.7)
HSIL/AIS	227 (44.4)	49 (28.3)	4 (36.4)	112 (53.3)	17 (23.0)	10 (58.8)	521 (21.6)	940 (27.5)
Carcinoma	15 (2.9)	11 (6.4)	0 (0.0)	4 (1.9)	1 (1.4)	1 (5.9)	5 (0.2)	37 (1.1)

^a^Including: Kangasala, Lempäälä, Nokia, Pirkkala and Ylöjärvi.

^b^Percentages represent the proportion within that specific genotype column, calculated based on the total number of women with available data for that section block (age, cytology, or histology).

^c^Age ranges represent pooled specific invitation birth cohorts (e.g., the 30–40 age group consists of women invited specifically at ages 30, 35, and 40).

Abbreviations: NILM = negative for intraepithelial lesion or malignancy; ASC-US = atypical squamous cells of undetermined significance; LSIL = low-grade squamous intraepithelial lesion; HSIL = high-grade squamous intraepithelial lesion; ASC-H = atypical squamous cells, cannot exclude HSIL; AGC-NOS = atypical glandular cells not otherwise specified; AGC-FN = atypical glandular cells, favor neoplasia; AIS = adenocarcinoma in-situ.

### Cumulative incidence and age-specific prevalence

The cumulative incidence of HSIL + histology for different HR-HPV genotype groups was evaluated across the age groups (Table 2). The analysis included all the referrals during the one screening round including women referred directly for colposcopy as well as those initially sent for retesting and later referred for colposcopy. HPV-negative women at the retesting were assumed to have normal histology^39^. The prevalence of HR-HPV genotypes varied by age, with HPV16 and HPV18 being most common among youngest women. Histological high-grade lesions were also more frequently observed in the youngest age group (n = 2550), where 26.2% (n = 669) of the HR-HPV positive women were diagnosed with histological HSIL + , whereas 15.9% (n = 191) in the 45–50-years-old age group (n = 1205) and 11.0% (n = 117) in the oldest age group (n = 1060) had HSIL + histology detected (*P* < 0.001). HPV16 and HPV18 positive women had higher cumulative incidence of histological high-grade lesions compared to the non-16/18-HPV types. Among the youngest age group, 46.5% (n = 186) of women with single HPV16 positivity (n = 400) had histological HSIL + detected, while this rate decreased to 18.4% (n = 21) in the oldest age group (n = 1149) (*P* < 0.001). Women with concurrent HPV16 and other HR-HPV infections at baseline had the highest risk of developing histological high-grade lesions.

**Table 2:** Cumulative incidence of histology confirmed HSIL + among the women invited in age groups of 30–40, 45–50 and 55–65 years that took part to the Finnish national cervical cancer screening between the year of 2012 to 2023 in the Tampere region and surrounding municipalities.

	Invitation age group: 30–40 years^c^ (n = 2550)		Invitation age group: 45–50 years^c^ (n = 1205)		Invitation age group: 55–65 years^c^ (n = 1060)		*P*-values^b^
HPV genotype	n (%)	Cumulative HSIL + n (%)^a^	n (%)	Cumulative HSIL + n (%)^a^	n (%)	Cumulative HSIL + n (%)^a^	
Single 16	400 (15.7)	186 (46.5)	135 (11.2)	35 (25.9)	114 (10.8)	21 (18.4)	**0.000**
Single 18	141 (5.5)	42 (29.8)	48 (4.0)	12 (25.0)	41 (3.9)	6 (14.6)	**0.148**
16/18	8 (0.3)	2 (25.0)	3 (0.2)	2 (66.7)	3 (0.3)	0 (0.0)	**0.184**
16 + othr	153 (6.0)	83 (54.2)	45 (3.7)	24 (53.3)	33 (3.1)	9 (27.3)	**0.017**
18 + othr	48 (1.9)	12 (25.0)	23 (1.9)	5 (21.7)	17 (1.6)	1 (5.9)	**0.240**
16/18 + othr	13 (0.5)	9 (69.2)	4 (0.3)	2 (50.0)	2 (0.2)	0 (0.0)	**0.171**
othr	1787 (70.1)	335 (18.7)	947 (78.6)	111 (11.7)	850 (80.2)	80 (9.4)	**0.000**
Any HPV	2550	669 (26.2)	1205	191 (15.9)	1060	117 (11.0)	**0.000**
*P*-values^b^		**0.000**		**0.000**		**0.004**	

Significant *P*-values are shown in bold.

^a^Percentage of the cumulative incidence in each HPV-genotype group.

^b^*P*-values showing the difference in cumulative incidence of HSIL + both between the three age groups and between the HPV genotype groups, using the chi-squared test

^c^Age ranges represent pooled specific invitation birth cohorts (e.g., the 30–40 age group consists of women invited specifically at ages 30, 35, and 40).

Abbreviations: othr = other high-risk HPVs (including 12 HR-HPVs of 31, 33, 35, 39, 45, 51, 52, 56, 58, 59, 66, 68); HSIL +  = high-grade squamous intraepithelial lesion or worse histopathology.

### Risk assessment by genotype and cytology

The association between histological HSIL + and different HR-HPV genotypes was further assessed, stratified by referral cytology outcomes (Table 3). First, looking at genotype risks irrespective of cytology, HPV16 infections, either alone or combined with other HR-HPV types, were significantly associated with a higher risk of developing histological HSIL + across all age groups with an OR of 3.47 (95%CI 2.89–4.17) and 5.74 (95%CI 4.43–7.43), respectively, when compared to women positive for the 12 other HR-HPV types. By contrast, HPV18 infections (single or combined) showed a weaker, though still significant, association with histological HSIL + with an OR of 1.91 (95%CI 1.45–2.50).

**Table 3:** Association between HR-HPV genotypes and risk of histology confirmed HSIL + , stratified by baseline cervical cytology and invitation age group, among women that took part to the Finnish national cervical cancer screening between the year of 2012 to 2023 in the Tampere region and surrounding municipalities.

Test combination		Invitation age group: 30–40 years^b^ (n = 2549)	Invitation age group: 45–50 years^b^ (n = 1204)	Invitation age group: 55–65 years^b^ (n = 1057)	All (n = 4810)
Cytology	HPV genotype	OR (95% CI)			
	Other HR-HPV (n = 3582)	1.00 (ref)	1.00 (ref)	1.00 (ref)	1.00 (ref)
- (n = 4810)	Single HPV16 (n = 648)	**3.77 (2.99–4.74)**	**2.66 (1.73–4.11)**	**2.20 (1.30–3.72)**	**3.47 (2.89–4.17)**
	HPV16 + other (n = 264)	**5.09 (3.69–7.02)**	**8.79 (4.92–15.69)**	**3.02 (1.38–6.61)**	**5.74 (4.43–7.43)**
	HPV18^a^ (n = 316)	**1.75 (1.25–2.45)**	**2.37 (1.33–4.23)**	1.36 (0.60–3.11)	**1.91 (1.45–2.50)**
NILM (n = 2594)	Other HR-HPV (n = 2090)	1.00 (ref)	1.00 (ref)	1.00 (ref)	1.00 (ref)
	Single HPV16 (n = 286)	**3.00 (1.98–4.54)**	2.21 (0.92–5.32)	0.71 (0.16–3.07)	**2.41 (1.55–3.75)**
	HPV16 + othr (n = 74)	**2.32 (1.08–4.97)**	**10.49 (3.59–30.68)**	3.72 (0.79–17.54)	**3.86 (2.14–6.96)**
	HPV18^a^ (n = 144)	1.34 (0.69–2.61)	2.14 (0.61–7.53)	NC	**2.03 (1.03–4.00)**
ASC-US (n = 984)	Other HR-HPV (n = 744)	1.00 (ref)	1.00 (ref)	1.00 (ref)	1.00 (ref)
	Single HPV16 (n = 133)	**2.58 (1.47–4.50)**	1.87 (0.70–4.96)	1.92 (0.58–6.36)	**2.54 (1.79–3.60)**
	HPV16 + othr (n = 55)	**4.33 (1.98–9.46)**	**4.78 (1.27–17.91)**	2.40 (0.61–9.47)	**4.49 (2.95–6.83)**
	HPV18^a^ (n = 52)	**3.33 (1.44–7.69)**	NC	1.92 (0.38–9.59)	**12.14 (7.04–20.92)**
LSIL (n = 505)	Other HR-HPV (n = 370)	1.00 (ref)	1.00 (ref)	1.00 (ref)	1.00 (ref)
	Single HPV16 (n = 57)	**4.07 (1.92–8.60)**	**5.08 (1.52–16.99)**	**15.56 (2.96–81.86)**	**5.19 (2.88–9.37)**
	HPV16 + othr (n = 46)	**3.50 (1.56–7.87)**	**9.48 (2.62–34.32)**	6.22 (0.86–44.95)	**4.93 (2.59–9.38)**
	HPV18^a^ (n = 32)	1.27 (0.44–3.64)	1.35 (0.15–12.52)	NC	1.24 (0.49–3.15)
HSIL (n = 504)	Other HR-HPV (n = 264)	1.00 (ref)	1.00 (ref)	1.00 (ref)	1.00 (ref)
	Single HPV16 (n = 133)	1.68 (0.89–3.16)	1.72 (0.58–5.09)	1.23 (0.32–4.81)	**1.83 (1.12–3.01)**
	HPV16 + othr (n = 70)	**2.75 (1.09–6.89)**	7.35 (0.88–61.23)	0.70 (0.09–5.44)	**3.16 (1.50–6.67)**
	HPV18^a^ (n = 37)	0.40 (0.16–1.00)	0.88 (0.24–3.22)	0.70 (0.09–5.44)	0.55 (0.27–1.10)
AGC (n = 223)	Other HR-HPV (n = 114)	1.00 (ref)	1.00 (ref)	1.00 (ref)	1.00 (ref)
	Single HPV16 (n = 39)	**3.33 (1.31–8.47)**	6.00 (0.48–74.29)	2.14 0.34–13.42)	**3.72 (1.73–7.97)**
	HPV16 + othr (n = 19)	2.38 (0.79–7.20)	3.00 (0.17–53.05)	NC	**2.86 (1.06–7.71)**
	HPV18^a^ (n = 51)	1.48 (0.62–3.51)	**5.25 (1.24–22.19)**	2.14 0.34–13.42)	**2.16 (1.10–4.25)**

Significant *P*-values are shown in bold.

^a^Includes both single HPV18 infections and HPV18 + other high-risk HPV infections.

^b^Age ranges represent pooled specific invitation birth cohorts (e.g., the 30–40 age group consists of women invited specifically at ages 30, 35, and 40).

Abbreviations: othr = other high-risk HPV (including 12 HR-HPVs of 31, 33, 35, 39, 45, 51, 52, 56, 58, 59, 66, 68); NILM = negative for intraepithelial lesion or malignancy (cytology); ASC-US = atypical squamous cells of undetermined significance (cytology); LSIL = low-grade squamous intraepithelial lesion (cytology); HSIL = high-grade squamous intraepithelial lesion including ASC-H (atypical squamous cells, cannot exclude HSIL) (cytology); AGC = atypical glandular cells (cytology); HSIL +  = high-grade squamous intraepithelial lesion or worse histopathology.

When stratifying by baseline cytology, distinct risk patterns emerged. Among women with NILM cytology, single HPV16 (OR 2.41, 95%CI 1.55–3.75), HPV16 coinfections (OR 3.86, 95%CI 2.14–6.96), and HPV18 infections (OR 2.03, 95%CI 1.03–4.00) all had a significantly higher risk of HSIL + compared to the 12 other HR-HPV types within the same NILM cytology group. Similarly, with ASC-US cytology, all HPV16/18 categories showed significantly increased risk compared to the other 12 HR-HPV types with ASC-US. Notably, the highest risk in this cytology group was observed for HPV18 infections with an OR of 12.14 (95%CI 7.04–20.92). Among women with LSIL cytology, single HPV16 and HPV16 coinfections maintained a high-risk relative to the other 12 HR-HPV types with LSIL (OR 5.19 and 4.93, respectively), while HPV18 infections did not show a significant difference in risk in this cytology category (OR 1.24, 95%CI 0.49–3.15).

### HR-HPV persistence and cumulative HSIL + detection

HR-HPV Persistence and Cumulative HSIL + Detection HR-HPV persistence was assessed among the 3337 women who had an initial HR-HPV positive result combined with mild cytology (NILM or ASC-US) and attended the recommended follow-up screening (Table 4). For this analysis, persistence was defined strictly as testing positive for the exact same partial HPV genotype group at the follow-up visit as detected at baseline. Only the youngest age group (30–40 years) showed significant differences in persistence rates between baseline genotype groups (*p* < 0.001). Among single HPV16 positives, 144 women (69.6%) were repeatedly persistently positive for single HPV16 at retesting, compared to 84.6% for those persistently HPV16 coinfections, and to 67.0% for persistently HPV18 infections. In contrast, 53.8% of women positive with the 12 other HR-HPV types had persistent infection with the same grouped genotype in the youngest age group. In the older age groups, persistence rates for single HPV16 infections were lower (57.5% and 57.7%, *p* = 0.057). Eventually, 22.0% of these HPV16-positive women and 12.9% of the HPV18-positive women with initial NILM or ASC-US cytology were diagnosed with histological HSIL + . To estimate the potential impact of immediate versus delayed referral for these high-risk groups, we calculated a hypothetical scenario where all 227 HPV16/18-positive women with mild cytology were referred directly to colposcopy. This strategy would have resulted in an approximate 7% increase in the total number of colposcopy referrals in this cohort, while detecting only one invasive carcinoma case earlier at the baseline screen rather than at follow-up.

**Table 4:** HR-HPV persistence rates^a^ among 3337 women attending follow-up screening after an initial HR-HPV-positive result with mild Pap cytology (NILM or ASC-US).

	Single HPV16		HPV16 + othr		HPV18*		Other HR-HPV		Any HR-HPV		*P-values*^b^
Invitation age group (years)^c^	n	Persistent n (%)	n	Persistent n (%)	n	Persistent n (%)	n	Persistent n (%)	n	Persistent n (%)	
30–40	207	144 (69.6)	65	55 (84.6)	103	69 (67.0)	1213	652 (53.8)	1588	920 (57.9)	**0.000**
45–50	87	50 (57.5)	23	16 (69.6)	40	21 (52.5)	706	349 (49.4)	856	436 (50.9)	**0.147**
55–65	78	45 (57.7)	30	23 (76.7)	43	26 (60.5)	742	419 (56.5)	893	513 (57.4)	**0.173**
*P-values*^b^		**0.057**		**0.273**		**0.264**		**0.026**		**0.002**	

Significant *P*-values are shown in bold.

*Includes both single and coinfections of HPV18.

^a^Persistent is defined as testing positive for the exact same partial HPV genotype group at the follow-up visit as at baseline (e.g., baseline HPV16 + must be HPV16 + at follow-up to be classified as persistent).

^b^"Any HR-HPV" column excluded from the chi-squared analysis across genotype groups.

^c^Age ranges represent pooled specific invitation birth cohorts (e.g., the 30–40 age group consists of women invited specifically at ages 30, 35, and 40).

As shown in the study flow chart (Fig. 1), 4646 women had baseline HR-HPV combined with NILM or ASC-US cytology; 1309 did not attend or were lost to follow-up, leaving a total of 3337 participants included in this analysis.

Abbreviations: othr = other high-risk HPV; ASC-US = atypical squamous cells of undetermined significance.

## Discussion

Tampere and its surrounding municipalities have been at the forefront of HPV primary screening in Finland, having implemented the program already in 2012^40^. With more than a decade of real-world experience, our findings underscore the value of incorporating HPV16/18 genotyping into screening protocols. Even partial genotyping enables more accurate risk stratification of women, supporting more tailored and effective clinical management.

The observed trend of decreasing overall HR-HPV prevalence by age, alongside HPV16 being consistently more prevalent than HPV18, aligns with findings from a global reviews and multinational studies^23,41–43^. Similar patterns have been reported in other Nordic cohorts historically unaffected by the HPV vaccination^44,45^. Study by Wang et al. evaluated numbers needed to screen (NNS) to prevent one cervical cancer case and found that with HPV16 and HPV18 NNS increased in older age groups suggesting better outcomes for younger women when screening these genotypes^45^. For the 12 other HR-HPV genotypes the NNS decreased with age. In the current cervical cancer screening algorithms in Finland, all 30- to 65-year-olds are screened with the same protocol. In line with other studies, our research suggests that different approaches could be considered for different age groups, with more vigorous follow-up protocols for younger women.

We observed that HPV16-positive women followed by HPV18 positive women had greater risk of high-grade cervical lesions compared to the 12 other HR-HPV positive women, which was in line with previous literature^19–22^. Although both HPV16 and HPV18 had a high risk for histology confirmed HSIL + , there was a substantial difference in the oncogenicity between these two genotypes in our cohort. Previous studies have also found that the risk of HPV18 and precancerous lesions does not differ considerably from the 12 other HR-HPV genotypes and separate triage for HPV18 should be evaluated in detail^46^. Also in the estimations of Wang et al. the effectiveness of follow-up strategies for HPV18 were said to need possible adjustments^45^. In addition, due to the high probability of HPV18 leading to adenocarcinomas, it must be considered that glandular lesions can often be missed in cytology-based reflex screening settings and even in colposcopies^47^.

Currently in Finland the women positive with the 12 other HR-HPVs and reflex cytology of LSIL are referred directly for colposcopy. In Norway, the recommendation to refer HPV16/18 positive women straight to colposcopy is based on “equal management for equal benchmark risk” -approach making sure that women at similar risks are treated similarly^18,48,49^. As in Norway, our results show that HPV16/18 positive women with NILM/ASC-US have similar or even higher risk of having HSIL + histology than women with LSIL cytology who are positive for the 12 other HR-HPV types. Therefore, a direct referral to colposcopy for all the HPV16/18 positive women, regardless of the cytology results, could be considered. Cytology results could be still used to determine a suitable urgency for the colposcopy appointment. However, it is worth noticing that referring these women straight to colposcopy would lead to an increase in total colposcopy referrals as shown in our results and, thus, slightly decreased screening specificity. Also, our results indicated that extremely few invasive carcinomas would have been detected earlier with this strategy. Consequently, avoiding unnecessary follow-up testing appears to be a more significant benefit of direct colposcopy referral than the potential advantage of achieving a faster diagnosis.

Large population-based study from Sweden showed that separate HPV genotyping can improve the national cervical cancer screening setting^45^. Currently in Finland all HR-HPV positive women with NILM or ASC-US cytology are referred for retesting in 12 to 24 months regardless of the HR-HPV genotype. Countries like Norway, Sweden and Denmark have conducted population level research and decided to refer all HPV16/18 positive women straight to colposcopy to avoid the unnecessary repeat screening after the next year when majority are still HPV positive^18,45,50^. Due to the high oncogenic potential of HPV16 and 18, even with NILM or ASC-US cytology the direct referral for colposcopy might be an effective strategy with the cost of some over-referring to colposcopy^51^. Our study showed that clear majority of the HPV16/18 positive women were HR-HPV positive also at retesting and thus eventually referred to colposcopy especially in the youngest age group. Over-referring to colposcopy could be mitigated by introducing straight colposcopy referral only for the younger age groups. Only two women in the oldest age group were diagnosed with carcinoma in our cohort and with the oldest age group, even extended screening intervals could be considered due to the less severe findings in colposcopy.

Looking forward, the entry of HPV-vaccinated cohorts into cervical cancer screening programs will profoundly impact screening strategies. As these cohorts, characterized by a significantly lower prevalence of HPV16 and HPV18, reach screening age, the relative epidemiological importance of non-16/18 HR-HPV genotypes will increase^52–55^. While the partial genotyping strategy assessed here is highly valuable in the current transitional phase, its utility may evolve as HPV16 and HPV18, currently the strongest markers of immediate risk, become rarer at the population level. Consequently, future screening protocols in Finland and other regions with HPV-vaccination will likely need to evaluate extended genotyping strategies that individually identify other potent carcinogenic types (e.g., HPV31, 33, and 45) to maintain effective risk stratification in the post-vaccination era.

The strength of this study was a real-world data from over ten years’ period and a large dataset including 76 482 routinely screened women. Cytology-level comparisons between different genotypes gives reliable information on different HR-HPV genotypes for risk stratification and opportunity to consider alternative cervical cancer screening strategies within the Finnish cervical cancer screening program. Regarding limitations, the study dataset included some women with deviations from the standard screening protocol (i.e., non-guideline referrals) and women lost to follow-up. However, these non-guideline cases were retained if sufficient data were available for analysis to reflect real-world clinical practice. Another limitation inherent to screening studies is verification bias; histological confirmation was only available for women referred to colposcopy. We assumed histological normality for HR-HPV negative women at follow-up, which may have slightly affected the results. Finally, due to sample size constraints, single HPV18 infections and HPV18 coinfections had to be combined for analysis.

To conclude, after more than a decade of HPV primary screening in Tampere and its’ surrounding municipalities, our findings support the implementation of partial HPV16/18 genotyping in Finland. The population risk profile aligns with that of other countries already using this approach. Separate triage for HPV16/18 positive women and age-specific screening strategies should also be considered. In addition, as HPV-vaccinated cohorts begin to enter screening, extended genotyping approaches should be further evaluated to optimize the Finnish cervical cancer screening program.

## Data Availability

Individual participant data that underlie the results reported in this article, after de-identification, will be available for researchers who provide a methodologically sound proposal with achievable aims. Proposals should be directed via e-mail to the corresponding Author Prof. Karolina Louvanto (karolina.louvanto@tuni.fi); data requestors will need to sign a data access agreement.
